# Genome‐Wide CRISPR‐Cas9 Screening Identifies NF‐κB/E2F6 Responsible for EGFRvIII‐Associated Temozolomide Resistance in Glioblastoma

**DOI:** 10.1002/advs.201900782

**Published:** 2019-07-24

**Authors:** Kai Huang, Xing Liu, Yansheng Li, Qixue Wang, Junhu Zhou, Yunfei Wang, Feng Dong, Chao Yang, Zhiyan Sun, Chuan Fang, Chaoyong Liu, Yanli Tan, Xudong Wu, Tao Jiang, Chunsheng Kang

**Affiliations:** ^1^ Tianjin Neurological Institute Key Laboratory of Post‐Neurotrauma Neuro‐Repair and Regeneration in Central Nervous System Ministry of Education and Tianjin City Tianjin 300052 China; ^2^ Department of Neurosurgery Tianjin Medical University General Hospital Tianjin 300052 China; ^3^ Beijing Neurosurgical Institute Department of Neurosurgery Beijing Tiantan Hospital Capital Medical University Beijing 100050 China; ^4^ Department of Cell Biology 2011 Collaborative Innovation Center of Tianjin for Medical Epigenetics Tianjin Key Laboratory of Medical Epigenetics Tianjin Medical University Tianjin 300070 China; ^5^ Department of Neurosurgery Affiliated Hospital of Hebei University Baoding 071000 China; ^6^ Department of Pathology Affiliated Hospital of Hebei University Baoding 071000 China

**Keywords:** CRISPR‐Cas9 libraries, E2F6, glioblastoma (GBM), temozolomide (TMZ) resistance

## Abstract

Amplification of epidermal growth factor receptor (EGFR) and active mutant EGFRvIII occurs frequently in glioblastoma (GBM) and contributes to chemo/radio‐resistance in various cancers, especially in GBM. Elucidating the underlying molecular mechanism of temozolomide (TMZ) resistance in GBM could benefit cancer patients. A genome‐wide screening under a clustered regularly interspaced short palindromic repeats (CRISPR)‐Cas9 library is conducted to identify the genes that confer resistance to TMZ in EGFRvIII‐expressing GBM cells. Deep sgRNA sequencing reveals 191 candidate genes that are responsible for TMZ resistance in EGFRvIII‐expressing GBM cells. Notably, E2F6 is proven to drive a TMZ resistance, and E2F6 expression is controlled by the EGFRvIII/AKT/NF‐κB pathway. Furthermore, E2F6 is shown as a promising therapeutic target for TMZ resistance in orthotopic GBM cell line xenografts and GBM patient‐derived xenografts models. After integrating clinical data with paired primary–recurrent RNA sequencing data from 134 GBM patients who received TMZ treatment after surgery, it has been revealed that the E2F6 expression level is a predictive marker for TMZ response. Therefore, the inhibition of E2F6 is a promising strategy to conquer TMZ resistance in GBM.

## 1 Introduction

Glioblastoma (GBM) is the most aggressive primary brain tumor with high proliferation and invasion and easy recurrence after surgery.^1^ Following advanced standard treatment, including resection followed by radio‐ and chemotherapy, the median survival time of GBM patients is only ≈14 months.^2^ Epidermal growth factor receptor (EGFR) is one of the important genes driving GBM. EGFR is amplified or mutated which leads to tumor cell invasion and tumor‐related angiogenesis via the aberrant activation of downstream signaling networks. EGFRvIII is the most common mutant that can be detected in up to 30% of GBM patients.^3^ EGFRvIII is derived from the deletion of exons 2–7, which results in an in‐frame deletion of 267 amino acids from the extracellular domain of wild‐type (wt) EGFR. EGFRvIII is incapable of binding any known ligands, but its tyrosine kinase is constitutively activated.

Although some new therapeutic strategies were emerging, such as systemic delivery of monoclonal antibodies,^4^ temozolomide (TMZ) is the oral alkylating agent that serves as the current standard therapeutic for newly diagnosed GBM. TMZ reportedly causes cell cycle arrest in the G2/M phase and mediates DNA damage and, subsequently, apoptosis.^5^ Although oral TMZ administration contributes to an overall increase in the survival of GBM patients, cancer cells eventually develop resistance to TMZ.^6^ Currently, overcoming TMZ resistance remains a major challenge for GBM treatment.

Searching for vulnerabilities of TMZ‐resistant GBM is a promising strategy to improve the therapeutic efficiency of TMZ. Here, we used a clustered regularly interspaced short palindromic repeats (CRISPR)‐Cas9 screening approach by applying a recently described GecKOv2 human library containing 123 411 sgRNAs that target 20 914 human genes, including 19 050 protein‐coding genes and 1864 microRNAs.^7^ We transduced U87 parental cells and U87EGFRvIII cells with lentiviruses of the pooled library. The sgRNA abundance was determined by deep sequencing upon the chemostress of TMZ. Among the candidate genes, we focused on E2F6 because its expression was increased in EGFRvIII‐overexpressing cells as determined by RNA‐seq. Furthermore, we found that E2F6 is a direct target gene of NF‐κB. We also demonstrated that E2F6 is a pivotal gene that mediates TMZ chemoresistance using gain‐ or loss‐of‐function experiments. Overall, our results suggest that E2F6 inhibition is a promising therapeutic strategy for TMZ‐resistant GBM.

## 2 Results

### 2.1 A Genome‐Wide Pooled sgRNA Library Screen Identifies Vulnerabilities in EGFRvIII‐Expressing GBM Treated with TMZ

**Figure 1:**
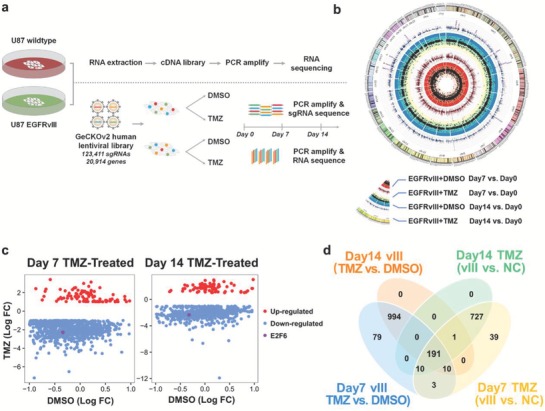
Vulnerabilities of TMZ identified by a genome‐wide pooled screening in EGFRvIII‐expressing GBM. a) Schematic representation of the flowchart of genome‐wide screening of EGFRvIII‐regulated/TMZ resistance‐associated genes using the pooled GeCKOv2 human lentiviral library. b) The sgRNAs of each gene were weighted, and 1287 and 1196 TMZ resistance‐associated genes were identified in U87EGFRvIII cells treated with TMZ at days 7 and 14, respectively (Table S5, Supporting Information). c) By comparing sgRNA profiles from the U87 and U87EGFRvIII cells following treatment with TMZ for 7 and 14 days, 981 and 929 differentially expressed genes were identified to be associated with TMZ resistance and sensitivity, respectively (Table S6, Supporting Information). d) Venn diagram shows differentially expressed genes in four groups described above: EGFRvIII‐expressing group (vIII), negative‐control group (NC), TMZ‐treated group (TMZ), and DMSO‐treated group (DMSO) (Table S7, Supporting Information).

**Figure 2:**
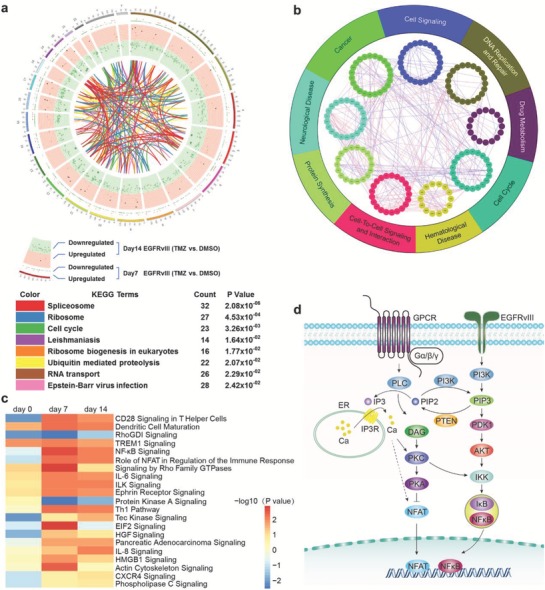
The major pathways involved in TMZ resistance. a) Differential expression profiles between EGFRvIII and control cells treated with TMZ at days 7 and 14. The KEGG database was used to show the connection between the genes (Table S6, Supporting Information). b) Differentially expressed genes were calculated by interactive genetic algorithm (IGA), and the genes were enriched in DNA replication, repair, and the cell cycle. c) Pathway enrichment analysis for up‐ and downregulated genes between EGFRvIII and control cells treated with TMZ at days 7 and 14. d) Schematics illustrating the EGFRvIII/PI3K/AKT/NF‐κB and GPCR/PLC/DAG/NFAT signaling pathways which are most significantly changed in EGFRvIII‐overexpressing cells treated with TMZ at two time points.

We first transduced U87 cells with lentiviruses expressing EGFRvIII and vector to obtain U87‐EGFRvIII and U87wt cells, respectively, which were used for genome‐wide pooled sgRNA screening. To identify the vulnerabilities of TMZ‐resistant GBM cells, we infected the U87‐EGFRvIII and U87wt cells with a sgRNA lentiviral library harboring 123 411 sgRNAs targeting 20 914 human genes, including 19 050 annotated protein‐coding genes and 1864 microRNA expression genes.^7^ The cells were treated with DMSO vehicle and TMZ for 7 and 14 days at 350 × 10^−6^ m, a concentration that predominantly inhibits U87wt cell proliferation. The coverage of sgRNAs in the cells was then calculated by high‐throughput sequencing after amplification of the sgRNA sequence in the genome (**Figure** **1**a). Compared with the untreated group, 1287 and 1196 genes in the U87EGFRvIII cells were identified as TMZ‐resistant candidates after treatment with TMZ for 7 and 14 days, respectively (Figure 1b). Further, we compared the U87wt and U87‐EGFRvIII libraries treated with TMZ for 7 and 14 days, and found 981 and 929 differentially enriched sgRNAs to be resistant or sensitive to TMZ, respectively (Figure 1c). Among these four groups, 191 sgRNAs were differentially enriched (Figure 1d). Kyoto encyclopedia of genes and genomes (KEGG) pathway analysis showed that these commonly differentially expressed sgRNAs were enriched in the spliceosome, ribosome, and cell cycle pathways (**Figure** **2**a). We further analyzed these genes by ingenuity pathway analysis (IPA) and demonstrated their association with cancer, the cell cycle, and the DNA repair pathway in U87‐EGFRvIII cells treated with TMZ for 14 days (Figure 2b; Table S2, Supporting Information). Compared with U87 cells, the most significantly altered pathways in U87‐EGFRvIII cells treated with TMZ were the CD28, NF‐κB signaling, and NFAT (Figure 2c; Table S3, Supporting Information). We simulated the pathways revealed by IPA and found that the EGFRvIII/PI3K/AKT/NF‐κB and G protein‐coupled receptor (GPCR)/PLA/PKC/NFAT pathways are the most responsible for TMZ resistance in EGFRvIII‐expressing GBM cells (Figure 2d; Table S4, Supporting Information).

### 2.2 E2F6 Was Induced by EGFRvIII and TMZ Treatment

To identify the vulnerabilities in EGFRvIII‐expressing cells that were resistant to TMZ treatment, we initially explored the genes possibly regulated by EGFRvIII through comparing the transcriptomic alterations between U87wt and U87‐EGFRvIII cells. Totally, 19 genes were upregulated in U87‐EGFRvIII cells with adjusted *P* value less than 0.05 and fold change larger than 2 (Figure S2a, Supporting Information). Subsequently, we screened the sgRNAs that were depleted in response to TMZ treatment for 14 days (Figure S2b, Supporting Information). We speculated that cells with overexpression of these genes showed therapeutic‐resistance to TMZ. Meanwhile, we conducted the RNA sequencing for U87‐EGFRvIII and U87wt cells under TMZ or DMSO treatment. Principal component analysis (PCA) showed that the TMZ‐induced differentially expressed genes were totally different from those in the DMSO‐treated counterparts (Figure S2c, Supporting Information). By overlapping the differentially expressed sgRNAs, mRNAs, and EGFRvIII‐regulated genes, E2F6 was the only hit induced by both the EGFRvIII mutation and TMZ treatment.

### 2.3 E2F6 Expression Is Correlated with Glioma Grade in Classical Subtype

**Figure 3:**
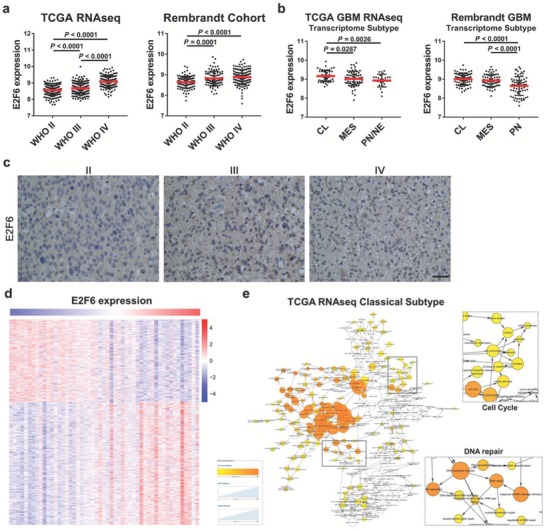
E2F6 expression is correlated with glioma grade in classical subtypes. a) Analysis of E2F6 expression in glioma TCGA RNA‐seq and Rembrandt cohorts. Elevated levels of E2F6 expression levels are associated with high grade of glioma. b) Analysis of E2F6 in three subtypes of glioblastoma, including classical (CL), mesenchymal (MES), and proneural (PN). E2F6 is enriched in patients with classical GBM. c) Representative IHC staining images of E2F6 in WHO II, III, and IV. Scale bar, 200 µm. d) Heatmap of E2F6‐associated genes in the TCGA RNA‐seq cohort. e) BiNGO pathway analysis of E2F6‐related genes.

To gain insight into the expression profile of E2F6 in glioma samples, we employed The Cancer Genome Atlas (TCGA) RNA‐seq data and microarray data of Rembrandt. Gliomas of World Health Organization (WHO) grade II, III, or IV were selected for analysis in our following study. As shown in **Figure** **3**a, the E2F6 expression level was significantly associated with tumor grade (*P* < 0.0001). Hence, the E2F6 expression level was higher in classical subtype than proneural (PN) and mesenchymal (MES) subtypes (Figure 3b, *P* < 0.003). In addition, analyses of TCGA HG‐U133A and Agilent 4502A microarrays and the RNA‐seq data cohorts revealed that the E2F6 expression was significantly elevated in GBM than normal brain tissues (NBT) (Figure S2d–f, Supporting Information). Furthermore, the immumohistochemical (IHC) staining of 31 WHO II patients, 32 WHO III patients, and 54 WHO IV patients proved that elevated levels of E2F6 expression are associated with high grade of glioma (Figure 3c). Additionally, E2F6 was found to be a valuable gene for GBM diagnosis with a high sensitivity and specificity in these three datasets (Figure S2g–i, Supporting Information). Thereafter, the correlated genes of E2F6 in the TCGA RNA‐seq data were selected (Figure 3d). KEGG analysis showed that these genes are associated with the cell cycle and DNA repair (Figure 3e).

### 2.4 E2F6 Is a TMZ‐Resistant Gene

**Figure 4:**
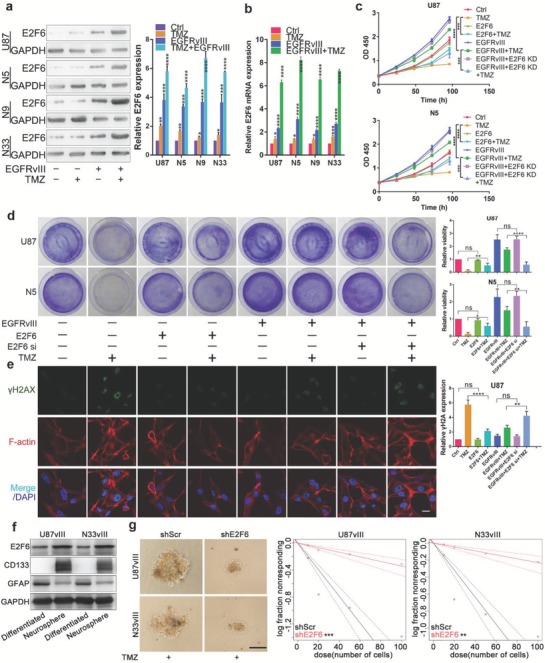
E2F6 is a key gene in TMZ resistance. a) Immunoblotting analysis of four GBM cell lines, which were infected and treated with and without EGFRvIII and TMZ, with indicated antibodies (left). E2F6 protein levels were quantified (right). (mean ± SD, *n* = 3, *P < 0.05, **P < 0.01, ***P < 0.001, ****P < 0.0001). b) Quantitative reverse transcriptase‐polymerase chain reaction (qRT‐PCR) analysis of E2F6 mRNA level in four cell lines infected and treated as described above. GAPDH was served as the negative control. (mean ± SD, *n* = 3, *P < 0.05, **P < 0.01, ***P < 0.001, ****P < 0.0001). c) Cell viability assay in U87 and N5 cell lines. The *P* value was calculated at 96 h. ***P < 0.001, ****P < 0.0001. d) Clonogenic assays were performed to reveal the effects of E2F6 on TMZ sensitivity in colony formation (left). Relative clones were calculated (right). (mean ± SD, *n* = 3, ns = no significance, *P < 0.05, **P < 0.01, ****P < 0.0001). e) Immunofluorescence staining of U87 cells with γ‐H2AX antibody after the cells were infected with EGFRvIII, E2F6, and/or siRNA/E2F6 and subsequently treated with and without TMZ. Cells overexpressing E2F6 resisted to TMZ‐induced DNA damage whereas depletion of E2F6 had opposite effects. Scale bar: 20 µm. f) Western blot analysis of E2F6, CD133, and GFAP levels in neurosphere and differentiated glioma cells. g) Representative images of spheres in dose of 100 cells per well are shown. Scale bar, 100 µm. In vitro limiting dilution assays culturing decreasing number of glioblastoma stem cells (GSCs) with or without E2F6 knockdown in U87vIII and N33vIII cells. The concentration of TMZ was 375 × 10^−6^ m. After 3 weeks, frequency and probability estimates were calculated using the ELDA software. **P < 0.01, ***P ≤ 0.001.

We next investigated the effects of E2F6 on TMZ resistance. Four GBM cell lines (U87, N5, N9, and N33) were stably infected with lentiviruses expressing EGFRvIII or vector control and were subsequently treated with TMZ or DMSO vehicle. Immunoblot analyses revealed that E2F6 level was significantly increased in EGFRvIII cells, as compared with vector‐infected GBM cells, and in the cells treated with TMZ (**Figure** **4**a,b), suggesting that E2F6 plays a pivotal role in TMZ resistance. Furthermore, we ectopically expressed and knocked down E2F6 in U87, U87‐EGFRvIII, N5, and N5‐EGFRvIII cells (Figure S3a,b, Supporting Information). Among the three small interfering RNAs (siRNAs) designed, siRNA#1 knocked down E2F6 with the greatest efficiency (E2F6 KD) and was therefore used in subsequent experiments (Figure S3c,d, Supporting Information). Following treatment with and without TMZ, the cells were subjected to CCK8 viability assay. We found that overexpression of E2F6 increased the resistance to TMZ, while silencing E2F6 reduced the resistance of EGFRvIII cells to TMZ (Figure 4c; Figure S3e, Supporting Information). Furthermore, the clonogenic assay also showed that E2F6 expression was closely correlated with TMZ resistance (Figure 4d). It has been shown that EGFRvIII confers radiation resistance by accelerating the repair of DNA double‐stranded breaks (DSBs).^8^ Because E2F6 was induced by EGRFvIII, we reasoned that E2F6 could increase the ability of DSB repair leading to TMZ resistance. To test this, we examined γ‐H2AX, a hallmark of DSB, level in U87, U87‐EGFRvIII, N5, and N5‐EGFRvIII cells after treatment with and without TMZ. Fluorescence staining revealed that DSB was induced upon the cells exposure to TMZ. Ectopic expression of either E2F6 or EGFRvIII dramatically reduced TMZ‐caused DSBs. However, knockdown of E2F6 largely abrogated the protective effect of EGFRvIII on DSBs (Figure 4e). Additionally, cancer stem cells are well recognized to be more resistant to TMZ. Thus, we assessed if E2F6 was highly expressed in neurosphere glioma cells. As shown in Figure 4f, E2F6 expression was significantly higher in neurosphere glioma cells than in differentiated glioma cells. The representative stem cell gene, CD133, was only detected in neurosphere glioma cells, and representative differentiated cell gene, GFAP, was downregulated in neurosphere glioma cells. We then performed a limiting dilution assay for sphere formation with two EGFRvIII‐expressing cell lines transduced with shE2F6 or shScr. We found the E2F6‐depleted U87vIII and N33vIII cells give rise to fewer and smaller spheres than the control cells in the context of 375 × 10^−6^ m TMZ treatment (Figure 4g). Taken together, these results demonstrate that E2F6 acts as a critical player by which EGFRvIII cells acquire resistance to TMZ by inhibition of DSBs.

### 2.5 E2F6 Is Regulated by NF‐κB in the EGFRvIII/PI3K/AKT Pathway

**Figure 5:**
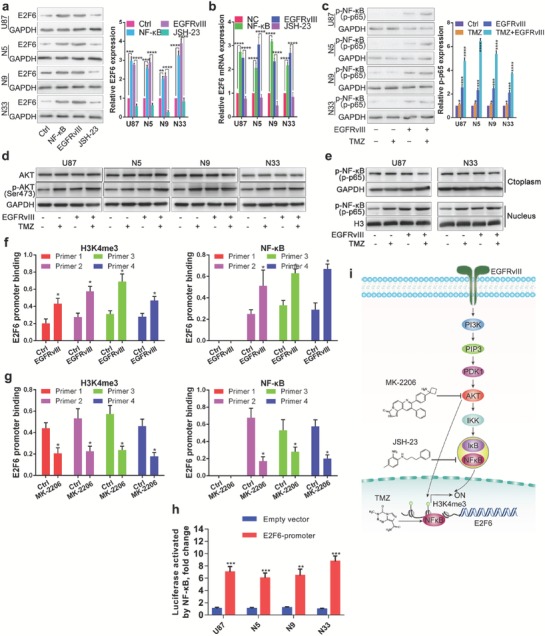
E2F6 is regulated by EGFRvIII/PI3K/AKT/NF‐κB and H3K4me3 modification. a) Western blot and b) qRT‐PCR analysis of E2F6 expression in GBM cells which were infected or treated with and without NF‐κB (p65/RelA), EGFRvIII, or JSH‐23. (mean ± SD, *n* = 3, ****P < 0.0001). c) Immunoblotting analysis of GBM cells with indicated antibodies after the cells were infected and treated with and without EGFRvIII and/or TMZ. p‐NF‐κB protein levels were quantified (right). (mean ± SD, *n* = 3, *P < 0.05, ***P < 0.001, ****P < 0.0001). d) Western blot analysis of AKT and p‐AKT (Ser 473) with indicated treatment in four GBM cell lines. e) The cytoplasmic fraction and the nuclear fraction were separated for Western blot analysis of p‐ NF‐κB P65. f,g) ChIP‐PCR assay: EGFRvIII increased the enrichment of H3K4me3 and p‐NF‐κB in E2F6 promoter (d) whereas AKT inhibitor MK‐2206 treatment reduced the interaction of H3K4me3 and p‐NF‐κB to E2F6 promoter (e). (*P < 0.05). h) Four GBM cell lines were cotransfected with Renilla plasmid, NF‐κB (p65/RelA) plasmid, and reporter vectors containing cloned ChIP‐qPCR fragments of the E2F6 promoters. Luciferase activity was normalized to Renilla. (mean ± SD, *n* = 3, **P < 0.01, ***P ≤ 0.001). i) Schematic illustration of the mechanism of E2F6 regulation by the EGFRvIII/PI3K/AKT pathway. NF‐κB was simultaneously activated by EGFRvIII/PI3K/AKT and TMZ, leading to transcriptional activation of E2F6. In addition, the EGFRvIII/PI3K/AKT induced H3K4me3 modification which further activated E2F6 transcription.

Previous studies showed that EGFRvIII preferentially activates phosphoinositide‐3‐kinase (PI3K)‐AKT,^9^ and subsequently noncanonical NF‐κB pathway.^10^ To identify whether E2F6 is regulated by AKT/NF‐κB pathway, we used MK‐2206 to inhibit AKT phosphorylation, JSH‐23 to inhibit nuclear translocation of NF‐kB (Figure S4a,b, Supporting Information). Subsequently, we treated the EGFRvIII cells with an AKT inhibitor MK‐2206 (10 × 10^−6^ m) for 48 h and found that the mRNA and protein levels of E2F6 were decreased (Figure S4c,d, Supporting Information). We next examined the effect of NF‐κB on E2F6 expression by treatment and transfection of GBM cells with NF‐κB inhibitor JSH‐23 (10 × 10^−6^ m) and RelA/p65 expression plasmid. Western blot and qRT‐PCR analyses revealed that the expression of E2F6 was positively associated with NF‐κB activation in GBM cells at the protein (**Figure** **5**a) and mRNA levels (Figure 5b). Additionally, NF‐κB and AKT were both activated by EGFRvIII and TMZ in total lysate of four GBM cell lines (Figure 5c,d). Furthermore, we separated the cytoplasmic fraction and the nuclear fraction and found translocation of the p‐NF‐kB to the nucleus was increased after EGFRvIII and TMZ treatment (Figure 5e). These observations suggest that E2F6 is induced by the EGFRvIII/PI3K/AKT/NF‐κB axis.

Moreover, we evaluated the chromatin state of the E2F6 promoter regulated by EGFRvIII in GBM cell lines. ChIP‐PCR analysis was performed using antibodies against H3K4me3 and p‐NF‐κB (p‐p65) and four pairs of genomic PCR primers specific for the E2F6 promoter (Figure S4e, Supporting Information). We found that the enrichment levels of H3K4me3 and p‐NF‐κB was increased at E2F6 promoter regions in EGFRvIII transfected GBM cells compared with the control cells (Figure 5f). Further, blocking AKT signaling using MK‐2206 abolished the enrichment of H3K4me3 and NF‐κB (Figure 5g). To further assess the functionality of the NF‐κB‐binding sites, we cloned ChIP‐qPCR fragments of the E2F6 promoters into the luciferase vector. GBM cells were cotransfected with RelA/p65 expression plasmid and reporter vectors. Luciferase test showed that NF‐κB directly activated E2F6 expression (Figure 5h). These data indicate that E2F6 transcription is regulated by NF‐κB downstream of the EGFRvIII/PI3K/AKT pathway (Figure 5i).

### 2.6 E2F6 Is a Promising Therapeutic Target for TMZ Resistance

**Figure 6:**
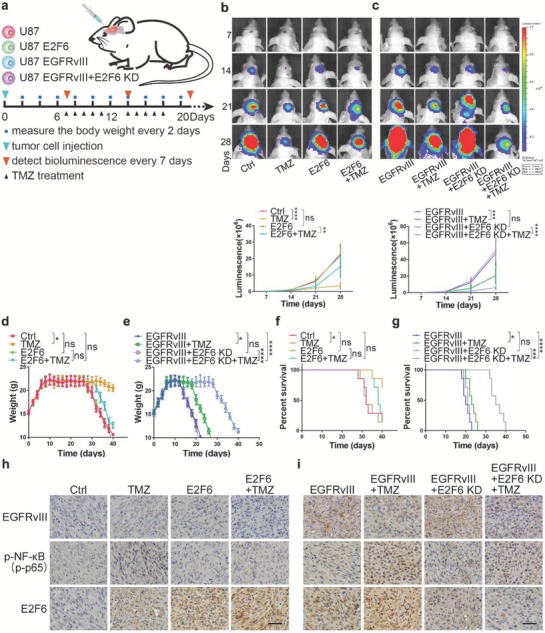
E2F6 is a causal factor of TMZ resistance in vivo. a) Schematic illustration of the evaluation of E2F6 as a causal factor in TMZ resistance in vivo. Mice were intracranially injected with U87, U87/E2F6, U87/EGFRvIII, or U87/EGFRvIII/E2F6 KD cells and subsequently treated by intraperitoneal injection of TMZ (5 mg kg^−1^ d^−1^) or DMSO for 2 weeks (5 days on and 2 days off) after being randomly divided into four groups (seven mice per group). Tumor growth was monitored by bioluminescence. b,c) Representative pseudocolor bioluminescence images from each group with indicated treatment underneath the images. Quantitative analysis of the photon flux is below. *P* value was calculated on day 28. (mean ± SD, *n* = 7, **P < 0.01, ***P < 0.001, ****P < 0.0001). d) The body weights of GBM‐bearing mice, which were established by intracranial injection of U87 and U87/E2F6 cells, and e) U87/EGFRvIII and U87/EGFRvIII/E2F6KD cells, after treatment with TMZ or vehicle control (*P < 0.05, ***P < 0.001, ****P < 0.0001, ns: not significant). f) Kaplan–Meier curve showed the overall survival time of GBM‐bearing mice, which were established by intracranial injection of U87 and U87/E2F6 cells, and g) U87/EGFRvIII and U87/EGFRvIII/E2F6KD cells, after treatment with TMZ or vehicle control (*P < 0.05, ***P < 0.001, ****P < 0.0001, ns: not significant). h) Representative immunostaining images of EGFRvIII, p‐NF‐κB, and E2F6 in the GBM xenografts, which were derived from U87 and U87/E2F6 cells, and i) U87/EGFRvIII and U87/EGFRvIII/E2F6KD cells, after treatment with TMZ or vehicle control. Scale bar: 50 µm.

Next, we investigated whether E2F6 acts as a critical factor in TMZ resistance in vivo. We stably infected U87 cells with lentiviruses expressing E2F6 or vector alone and U87EGFRvIII cells with a lentivirus encoding E2F6 siRNA#1 (E2F6 KD) or vector alone. Orthotopic GBM mouse models were constructed by intracranially injecting these four groups of cells into mice. 1 week later, the mice were intraperitoneally injected with DMSO or TMZ (5 mg kg^−1^ d^−1^) for 2 weeks at 5 days on and 2 days off (**Figure** **6**a). The bioluminescence imaging analyses showed that the tumors established with U87/E2F6 cells resisted TMZ treatment (Figure 6b), whereas the tumors created with U87EGFRvIII/E2F6siRNA cells were sensitive to TMZ (Figure 6c), when compared to the tumors established with their vector control cells. Moreover, we observed that TMZ treatment significantly reduced the body weight lost in vector infected U87 and U87/EGFRvIII mice, especially in U87/EGFRvIII/E2F6KD mice when compared to untreated mice (Figure 6d,e), but had no notable effect on U87/E2F6 mice (Figure 6d). Notably, Kaplan–Meier survival curve analysis showed that TMZ treated U87 and U87/EGFRvIII/E2F6KD mice had much longer survival time than untreated mice (Figure 6f,g), but had no notable effect on U87/E2F6 (Figure 6f). These findings indicate that E2F6 plays a pivotal role in TMZ resistance in vivo. Immunohistochemical staining of xenografts revealed that tumors derived from U87 cells treated with TMZ exhibited elevated levels of p‐NF‐κB and E2F6 (Figure 6h). Moreover, p‐NF‐κB and E2F6 were significantly increased in EGFRvIII‐bearing tumors, especially the tumors treated with TMZ (Figure 6i). Taken together, these data showed that E2F6 is a vulnerability related to TMZ‐resistant GBM and that inhibition of E2F6 could be a promising therapeutic strategy for TMZ‐resistant GBM.

### 2.7 Inhibition of NF‐κB/E2F6 Axis Sensitizes GBM to TMZ

**Figure 7:**
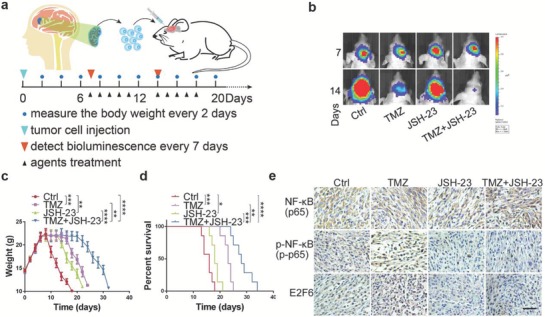
JSH‐23 treatment enhances the therapeutic efficacy of TMZ. a) Schematic illustration of the evaluation of the therapeutic efficacy of TMZ or JSH23 alone and their combination in vivo. Mouse GBM models were established by transplanting primary GBM cells isolated from fresh GBM samples into mouse brain. Mice bearing GBM were intraperitoneally treated with TMZ (5 mg kg^−1^ d^−1^), JSH‐23 (6 mg kg^−1^ d^−1^), TMZ (5 mg kg^−1^ d^−1^)/JSH‐23 (6 mg kg^−1^ d^−1^), or PBS vehicle control for 2 weeks (5 days on and 2 days off). Tumor growth was evaluated by bioluminescence once a week, and the mouse body weights were measured every 2 days. b) Representative bioluminescence images of the intracranial PDX mice treated with indicated agents at days 7 and 14 (mean ± SD, *n* = 7, **P < 0.01, ***P < 0.001, ****P < 0.0001). c) Body weights of PDX mice treated with indicated agents (*P < 0.05, **P < 0.01, ***P < 0.001, ****P < 0.0001). d) Kaplan–Meier curve shows that the overall survival time was increased in TMZ/JSH‐23 combination treatment group (**P < 0.01, ***P < 0.001, ****P < 0.0001). e) Representative immunostaining results of expression of NF‐κB, p‐NF‐κB, and E2F6 in PDX tumors after treatment with indicated agents. Scale bar: 50 µm.

Because there is no small molecule inhibitor of E2F6 available, we first assessed whether p‐AKT inhibitor (MK‐2206) and NF‐κB inhibitor (JSH‐23) had synergistic effect with TMZ treatment. Compared with JSH‐23 or TMZ alone, JSH‐23 plus TMZ exhibited enhanced cytotoxicity in GBM cells. The confidence interval (CI) values were all <0.8, indicating a strongly synergistic interaction between JSH‐23 and TMZ in GBM cells (Figure S5a, Supporting Information). Similarly, MK‐2206 also has synergistic effect with TMZ treatment in GBM cells (Figure S5b, Supporting Information). Subsequently, we investigated whether pharmacological inhibition (JSH‐23) of NF‐κB, a critical regulator of E2F6, inhibits tumor growth and sensitizes TMZ in orthotopic patient‐derived GBM xenograft mouse model. Tumor samples from primary GBM following three courses of TMZ treatment from a patient were orthotopically injected into mice. This patient is also a patient with a recurrent GBM after surgery and TMZ treatment. After 1 week, the mice were randomly divided into four groups (seven mice per group) which were treated with TMZ (5 mg kg^−1^ d^−1^), JSH‐23 (6 mg kg^−1^ d^−1^), TMZ (5 mg kg^−1^ d^−1^)/JSH‐23 (6 mg kg^−1^ d^−1^) and phosphate buffered saline (PBS) vehicle control for 2 weeks at 5 days on and 2 days off (**Figure** **7**a). Bioluminescent imaging revealed that the mice treated with a combination of TMZ and JSH‐23 exhibited sustained tumor regression compared with mice treated with either one alone (Figure 7b). In addition, the combination treatment group significantly reduced body weight loss rate (Figure 7c) and had a longer survival time when compared with the other groups (Figure 7d). On day 14 of the treatment, the representative mice from each group were euthanized and the xenograft tumor was removed for immunohistochemistry analysis. We found that that tumors treated with TMZ alone expressed elevated levels of p‐NF‐κB and E2F6, whereas JSH‐23 treatment inhibited p‐NF‐κB and E2F6 expression (Figure 7e). Taken together, these data indicate that suppression of E2F6 by inactivation of NF‐κB could increase TMZ sensitivity.

### 2.8 E2F6 Is a Poor Prognostic Marker in GBM

**Figure 8:**
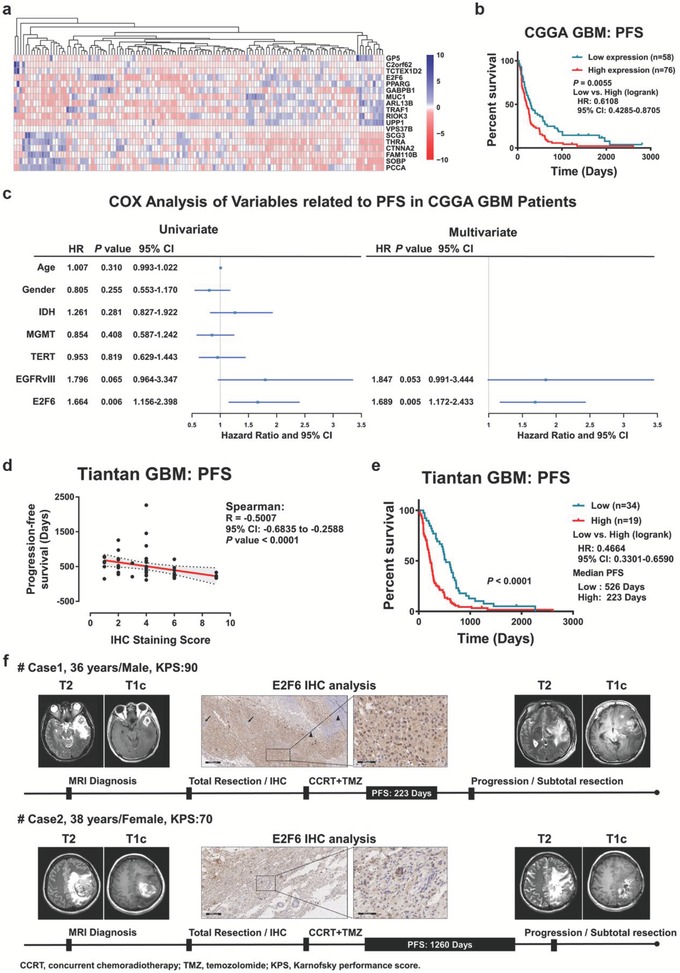
E2F6 is a valuable biomarker in clinical GBM samples. a) Univariate Cox analyses showed that 18 of the 191 candidate genes were significantly associated with the PFS in the CGGA GBM cohort. b) Kaplan–Meier analysis revealed that E2F6 expression level was negatively correlated to the PFS in patients with GBM (*n* = 134, *P* = 0.0055). The cutoff of E2F6 was identified using X‐tile software. c) Univariate and multivariate Cox analyses showed that E2F6 is an independent prognostic marker in the CGGA GBM cohort. d,e) Immunohistochemical staining GBM tumors from Tiantan Hospital with E2F6 antibody. Expression levels of E2F6 were negatively associated with the PFS (Spearman's correlation: *R* = −0.5007, *P* < 0.0001). f) Representative magnetic resonance images, E2F6 immunochemistry, and progression‐free survival data in two GBM patients.

To further evaluate E2F6 as a biomarker in GBM patients, we collected 134 GBM samples from the Chinese Glioma Genome Atlas (CGGA) RNA‐seq database with PFS (progression‐free survival) data available. Among the 191 candidate genes, univariate Cox analyses revealed that 18 PFS‐associated genes include E2F6, MUC1, and TRAF1 (**Figure** **8**a). High level of E2F6 was significantly correlated with poor PFS (Figure 8b). Further analysis showed that E2F6 may serve as an independent poor prognostic marker (Figure 8c; hazard ratio: 1.689, 95% confidence interval: 1.172–2.433, *P* = 0.005). Moreover, we analyzed the relationship between the E2F6 protein level and PFS in 53 GBM patients (Primary tumors) who had undergone standard treatment in Beijing Tiantan Hospital. Immunohistochemical staining analysis and statistical analyses revealed that E2F6 expression level was negatively related to PFS (Figure 8d), i.e., patients whose tumors express high levels of E2F6 had a significantly short PFS (Figure 8e,f).

## 3 Discussion

In this study, we performed a genome‐wide screening in GBM cells treated with and without EGFRvIII and TMZ and identified the PI3K/AKT/NF‐κB pathway responsible for TMZ resistance in EGFRvIII‐expressing GBM cells. More significantly, EGFRvIII rendered GBM cells to TMZ resistance and E2F6 was identified as a critical target in TMZ resistance and GBM bearing EGFRvIII. TMZ treatment and EGFRvIII induce E2F6 at both protein and mRNA levels through activation of the PI3K/Akt/NF‐kB pathway. Depletion of E2F6 sensitizes GBM cells to TMZ and abrogates EGFRvIII‐associated TMZ resistance. These findings are important in several folds: first, demonstration of E2F6 as a pivotal downstream target gene of EGFRvIII/ PI3K/AKT/NF‐κB axis; second, established direct connection of E2F6 with EGFRvIII‐promoted GBM progression and TMZ resistance; and finally, provided evidence of E2F6 as a potential therapeutic target and a valuable prognostic marker for GBM, especially the tumors harboring EGFRvIII.

Currently, TMZ is the most commonly used and most effective drug in the treatment of GBM. TMZ administration alone can improve the median survival of GBM patients by 8 months.^11^ However, almost all patients eventually develop resistance. Moreover, recurrent GBMs are generally more aggressive than primary tumors. Therefore, there is a critical need to understand how TMZ resistance is acquired.

TMZ mediates cytotoxicity primarily via the formation of an O6‐methylated guanine (O6MeG) adduct, which causes the attachment of thymine‐substituted cytosine residues to O6‐methylated guanine during DNA replication. This mismatch repair injury leads to replication‐associated DNA DSBs, often triggering cell death.^12^ The increase in the expression of O6‐methylated guanine‐DNA methylase (MGMT) has been shown to cause the resistance, which makes the tumor resistant to TMZ via repair of the O6 guanine adduct.^13^ In addition, growing evidence suggests that other proteins mediate tumor resistance to TMZ. A large body of literature has demonstrated that the PI3K/AKT pathway plays an important role in TMZ resistance,^14^ and evidence exists that glioma is resistant to TMZ via the PI3K/AKT/NF‐κB signaling cascade.^15^ Especially, activation of NF‐κB is a general cellular response to anticancer drugs.^16^ Therefore, the treatment of TMZ is likely to also activate NF‐κB through some mechanisms. In this report, we showed that EGFRvIII rendered GBM cells resistant to TMZ by activating the PI3K/AKT/NF‐κB pathway. Furthermore, we employed a patient‐derived xenografts (PDX) mouse model to reveal that inactivation of NF‐κB is a promising therapeutic strategy to increase TMZ sensitivity. Recently, the bortezomib, a therapeutic proteasome inhibitor,^17^ in combination with TMZ lacked efficacy in advanced refractory solid tumors or melanoma patients in a phase II clinical trial (Clinicaltrials.gov identifier: NCT00512798). Although bortezomib and JSH‐23 both inhibited NF‐κB in different ways, the key downstream factors are distinct. The inhibitory effects of bortezomib on cell growth are potentially because of downregulation of cytokines CXCL8 and CXCL1, which are regulated by NF‐κB and play an important role in promoting growth and metastasis of melanomas.^17^ However, CXCL8 and CXCL1 maybe are not the TMZ resistance genes, so the combined effect with TMZ might just be synergy of toxicity, not chemotherapy sensitization. It is recognized that activation of NF‐κB is associated with drug résistance in many tumors, but we believe there should be other critical gene‐mediated drug resistance. In this study, we identified E2F6 as a key gene associated with TMZ resistance in EGFRvIII GBM using the CRIPSR‐Cas9‐based genome‐wide screening system. What's more, E2F6 expression is controlled by the EGFRvIII/AKT/NF‐κB pathway. Thus, combination with JSH‐23 and TMZ should be a promising treatment for EGFRvIII GBM or EGFR amplification GBM or classical subtype GBM.

Previous studies have shown that the NF‐κB is activated by both canonical and noncanonical pathways. As a noncanonical pathway, EGFRvIII activates NF‐κB via PI3K/AKT. In addition, NF‐κB may also be activated by DNA damage response and is thought to play a key role in cell survival.^18^ Many genotoxic drugs also induce both NF‐κB and canonical and noncanonical signaling pathways.^19^ In addition, NF‐κB is activated by TMZ‐induced mismatch repair and AKT‐dependent activation protects cells from cytotoxicity.^15^ The role of DNA damage‐induced NF‐κB activation in cell protection or apoptosis induction depends on the cell line and dose and drug characteristics.^19^ In the present study, we found the NF‐κB activation induced by EGFRvIII and TMZ to promote GBM cell survival and TMZ resistance. Notably, we identified that E2F6 is transcriptionally activated by NF‐κB. In addition, EGFRvIII/AKT increases the accumulation of H3K4me3 in the E2F6 promoter region, thereby further enhancing E2F6 expression at the transcriptional level.

The E2F6 protein is an Rb‐independent transcriptional repressor. E2F6 inhibits E2F‐dependent S‐phase transcription and provides transcriptional conditions for G1/S‐related genes.^20^ Studies have shown that due to replication pressure, Chk1 maintains E2F1‐3 transcriptional activity via the phosphorylation of E2F6.^21^ Previous studies showed that E2F6, E2F7, and E2F8 are induced in cells treated with a DNA‐damaging agent and play important roles in DNA damage repair, increasing cell resistance to chemotherapeutic drugs and maintaining cell survival.^22^ E2F6 has been recently shown to promote cancer stem cell survival and growth and to be frequently overexpressed in a number of cancers.^23^ Here, using CRISPR based genome‐wide screening we identified E2F6, but not other members of E2F family, as an important TMZ‐resistant gene in EGFRvIII‐expressing GBM cells. TMZ‐induced DNA damage activates NF‐κB, resulting in significantly increased E2F6 expression. Elevated E2F6 abrogates the inhibitory effect of TMZ.

In summary, our study provides a strategy to identify the genetic determinants of TMZ resistance in GBM using the CRIPSR‐Cas9‐based genome‐wide screening system, which leads to uncover additional promising therapeutic targets for GBM patients. We demonstrated that E2F6 plays a critical role in TMZ resistance in GBM, especially GBM harboring EGFRvIII. E2F6 was induced by EGFRvIII and TMZ through the noncanonical NF‐κB pathway. Depletion of E2F6 largely abrogates EGFRvIII‐associated TMZ resistance. These findings suggest that inhibition of E2F6 is a plausible strategy for the development of therapies and that an assessment of E2F6 expression could be useful as a predictive biomarker.

## 4 Experimental Section

*Cell Culture, Lentiviruses, and Chemicals*: The human glioblastoma U87 cell line was purchased from ATCC (American Type Culture Collection, Manassas, VA, USA) and maintained in a humidified 5% CO_2_ atmosphere at 37 °C. Primary cell lines (N5, N9, and N33) were derived from GBM patients who underwent surgery at Tiantan Hospital according to a protocol reported by Dong et al.^24^ EGFRvIII cell lines were generated via infection of the cells with lentiviruses expressing EGFRvIII (GV341‐EGFRvIII) followed by puromycin selection. The cells were routinely maintained in Dulbecco's Modified Eagle's Medium (DMEM) with 10% heat‐inactivated fetal bovine serum (FBS, HyClone, Logan, UT, USA) in a humidified 5% CO_2_ atmosphere at 37 °C. Lentiviruses encoding the E2F6 protein and E2F6 siRNA‐1, E2F6 siRNA‐2, or E2F6 siRNA‐3 were obtained from Genechem (Shanghai Genechem Co., Ltd., Shanghai, China). The sequences of E2F6 siRNAs were: E2F6 siRNA‐1: 5′‐GAGGAACTTTCTGACTTAT‐3′; E2F6 siRNA‐2: 5′‐ATGTCTATTTGTGTGAAGT‐3′, and E2F6 siRNA‐3: 5′‐ACTTAGATTACTGAGTAAT‐3′. TMZ and puromycin were obtained from Sigma‐Aldrich (St. Louis, MO, USA), while MK‐2206 and JSH‐23 were obtained from Selleck Chemicals (Houston, TX, USA).

*Pooled Genome‐Wide CRISPR Screening*: U87 and U87‐EGFRvIII cells were treated with a series of concentrations of TMZ (Figure S1a,b, Supporting Information). At 375 × 10^−6^ m concentration, TMZ inhibited cell growth much more significantly in U87 than U87‐EGFRvIII cells (Figure S1c, Supporting Information). Therefore, 375 × 10^−6^ m TMZ was chosen in the following experiments. The U87 and U87‐EGFRvIII cells were infected with the pooled GeCKOv2 human lentiviral library at a multiplicity of infection (MOI) of 0.3 to ensure that most cells received only one stably integrated RNA guide. On day 2, puromycin was added to the cells to select the positively transduced cells. On day 9, the cells were treated with 375 × 10^−6^ m TMZ. After 7 and 14 days of TMZ treatment, the cells were harvested, and genomic DNA was isolated for the amplification of sgRNAs, which were then sequenced on an Illumina X instrument. The sequences data were aligned to the sgRNAs using Bowtie 2 (GSE 112733).

*Quantitative RT‐PCR*: Total RNA was isolated using TRIzol Reagent (Invitrogen, Carlsbad, CA, USA) following the manufacturer's instructions. cDNA library was constructed by reverse transcription using the GoScript Reverse Transcription System (Promega, Madison, WI, USA) according to the manufacturer's protocol. The cDNA was amplified using SYBR Green Master Mix (Applied Biosystems/Thermo Fisher, Austin, TX, USA) and normalized by glyceraldehyde 3‐phosphate dehydrogenase (GAPDH). The experiments were performed in triplicate on a CFX96 PCR cycler (Bio‐Rad, Hercules, CA, USA). The primer sequences used are listed as below.

E2F6 Forward: 5′‐TCAGCAAAGTGAAGAATTGC‐3′
E2F6 Reverse: 5′‐CGAGAGCACTTCATGGATAA‐3′
GAPDH Forward: 5′‐TTGGTATCGTGGAAGGACTCATG‐3′
GAPDH Reverse: 5′‐GTTGCTGTAGCCAAATTCGTTGT‐3′

Relative gene expression was calculated as the 2^−ΔΔCt^ fold change.

*RNA Sequencing*: Total RNA was isolated as described above for RNA‐seq analysis. The cDNA library was generated and amplified by PCR. For RNA‐seq of EGFRvIII‐overexpressing cells, the cDNA library was sequenced using Illumina HiSeq4000, and sequence data were aligned to the hg19 reference using Hisat2 (GSE 112734). For TMZ‐treated cells, U87 and U87‐EGFRvIII were individually treated with TMZ or DMSO for 7 and 14 days. The cDNA library was constructed, and sequencing was performed by the Beijing Genomics Institute on the BGISEQ‐500 platform. Sequence data were aligned to the hg19 human genome reference using Bowtie2 (GSE 112735). The expression levels of the genes were measured by fragments per kilobase of exon model per million mapped reads (FPKM).

*Western Blot Analysis*: Western blot analysis was performed as previously described.^25^ Briefly, cells were lysed with radio immunoprecipitation assay (RIPA) buffer (Solarbio, Beijing, China) for total lysate extraction. The Nuclear Protein Extraction Kit (Solarbio, Beijing, China) was used to extract the cytoplasmic fraction and the nuclear fraction. Then, equal amounts of proteins were subjected to 10% sodium dodecyl sulfate‐polyacrylamide gel electrophoresis (SDS‐PAGE) and transferred to a polyvinylidene difluoride membrane. The membrane was first blocked by 5% bovine serum albumin (BSA) for 1 h at room temperature, followed by incubation at 4 °C overnight with the following primary antibodies: p‐NF‐κB (Cell Signaling Technology (CST), Boston, MA, USA, 1:1000), E2F6 (GeneTex, Irvine, CA, USA, 1:1000), and GAPDH (Millipore, Billerica, MA, USA, 1:2000). After washing three times with phosphate buffered saline‐Tween 20 (PBST), the membrane was incubated with antimouse and antirabbit horseradish peroxidase‐conjugated secondary antibodies (Promega, 1:10 000) for 1 h at room temperature. Immunoblots were developed using G:BOX F3 (Syngene, Cambridge, UK).

*Clonogenic Assay*: Cells (2 × 10^3^ per dish) were seeded in 60 mm Petri dishes and cultured for 21 days. Subsequently, the cells were washed twice with PBS, fixed with methanol and acetic acid at a 3:1 ratio, and then stained with 6% v/v glutaraldehyde and 0.5% crystal violet (Solarbio) for 5 min at room temperature. The plates were thoroughly washed with water and air‐dried at room temperature. The number of colony was accounted.

*Cell Viability Assay*: Cell viability assay was conducted using cell counting kit‐8 (CCK‐8 Kit) (Dojindo Laboratories, Kumamoto, Japan) according to the manufacturer's instructions. Briefly, the cells (2 × 10^3^ cells per well) were cultured in 96‐well plates in triplicate. After allowing the cells to attach to the bottom of the plate for 12 h, the cells were treated with TMZ or DMSO vehicle for 0–96 h. At the indicated time, 10 µL of the CCK‐8 solution was added to each well, and the absorbance of the converted dye was measured using a microplate reader (Synergy2, BioTek, USA).

*ChIP and ChIP‐qPCR Assays*: ChIP was performed using a ChIP Assay Kit (Millipore) according to the manufacturer's instructions. Briefly, after fixing with 1% formaldehyde for 10 min and neutralizing with glycine for 5 min at room temperature, 1 × 10^6^ mL^−1^ cells were washed with cold PBS, scraped, and stored on ice. The cells were then resuspended in a ChIP lysis buffer and sonicated 90 times using the high‐power setting of the 3 s on/9 s off sonication cycle in ice water with a Sonics Vibra‐Cell processor (Sonics & Materials Inc., Newtown, CT, USA) to generate 300 to 1000 bp DNA fragments. In total, 10% of the lysate was used as the DNA “input” control. The remaining samples were immunoprecipitated with antibody‐coupled magnetic beads on a rotator at 4 °C overnight. The following antibodies were used: anti‐p‐NF‐κB (p‐p65) (CST), anti‐H3K4me3 (CST), and normal mouse immunoglobulin G (IgG) (Millipore). The immunoprecipitates were collected using a magnetic rack. The beads were washed and bound chromatin was eluted in a ChIP Elution Buffer. The chromatin products were treated with RNase A (15 min at 37 °C) and proteinase K (2 h at 55 °C). After DNA purification, the E2F6 promoter binding site was quantified using qPCR and normalized by total chromatin (input). Normal mouse IgG was used as a negative control, and the primers used are listed as below.

1# Forward: 5′‐CGGTGTGTTGCCTTTTTATT‐3′
1# Reverse: 5′‐AACAACGTCCAATTTCAGTG‐3′
2# Forward: 5′‐CACTGAAATTGGACGTTGTT‐3′
2# Reverse: 5′‐AGGTCAGTGTTGATGCTTAG‐3′
3# Forward: 5′‐ATCTCTGCGGCTCAGAACTT‐3′
3# Reverse: 5′‐AGGGAACAGGGGTGAGAGAA‐3′
4# Forward: 5′‐TTTCCTTTGCCAGCCTCTCC‐3′
4# Reverse: 5′‐ACGCAGACGGAAAAAGAGGA‐3′

*Chromatin Immunoprecipitation Sequencing (ChIP‐Seq)*: Density maps were generated with read extension to 200 bp with IGVtools based on the hg19 human reference genome. The ChIP‐seq data for GM12878 cells modified by H3K4me1, H3K4me3, H3K27ac, and NF‐κB were obtained as interactive tracks from the UCSC Genome Browser (http://genome.ucsc.edu/).

*Immunohistochemistry*: Immunohistochemistry was performed on 5 µm thick formalin‐fixed, paraffin‐embedded (FFPE) sections, and the sections were deparaffinized in xylene and rehydrated in ethanol. Antigen retrieval was performed with a citrate‐buffered solution at 95 °C for 15 min, and the sections were stained with antibodies against E2F6 (GeneTex, 1:100), EGFRvIII, and p‐NF‐κB (CST, 1:100) at 4 °C overnight. After washing with PBS (pH 7.4) three times for 5 min each, the primary antibody was detected using a ZLI‐9023 kit (ZSGB‐BIO, Beijing, China), and nuclear counterstaining was performed with hematoxylin for 10 min. The slides were analyzed using National Institutes of Health (NIH) ImageJ software. The scores of the E2F6 IHC analyses were determined by combining the proportion of positively stained tumor cells and the intensity of the staining. The positivity of stained tumor cells was graded as follows: I, <5% positive tumor cells; II, 5–20% positive tumor cells; and III, >20% positive tumor cells. The intensity of the staining was recorded on a scale of 1 (weak staining), 2 (moderate staining), and 3 (strong staining). The staining index was calculated as follows: staining index = staining intensity × the percentage of positive cells. High E2F6 expression was defined as a staining index score ≥6.

*Clinical Specimens*: In total, 53 pairs of glioma samples (primary and recurrent tumors) who had subjected to standardized TMZ treatment were collected from Tiantan Hospital. Clinical data were collected (Table S1, Supporting Information). Formalin‐fixed, paraffin‐embedded tumor specimens were prepared for immunohistochemistry study. The samples were processed in accordance with ethical standards of the 2008 Helsinki Declaration. All patients provided written consent for the use of their samples for biomedical research. The tumors were histologically subtyped and graded according to the 2007 World Health Organization classification of nervous system tumors. Clinical data in Figure 3c were collected (Table S9, Supporting Information).

*Patients and Samples for Microarray Data*: HG‐U133A, Agilent‐4502A microarray, and RNA‐seq data from The Cancer Genome Atlas were downloaded from the University of California Santa Cruz (UCSC) Cancer Genome Browser (https://genome‐cancer.ucsc.edu). The relevant clinical and molecular information were obtained from the TCGA database (https://tcga‐data.nci.nih.gov/docs/publications/lgggbm_2015/).^26^ Microarray data for validation were obtained from the US National Cancer Institute Repository for Molecular Brain Neoplasia Data (REMBRANDT: https://gdoc.georgetown.edu/gdoc) cohort (*n* = 313).^27^ RNA‐seq data were acquired from the Chinese Glioma Genome Atlas database (http://www.cgcg.org.cn/, *n* = 325).^28^ RNA‐seq data with progression‐free survival information in patients treated with TMZ after the first surgery were obtained from the CGGA database (*n* = 134).

*Neurosphere‐Forming Assay and Limiting Dilution Analysis*: Neurosphere‐forming assay was performed in low attachment six‐well plates (Corning). U87EGFRvIII and N33EGFRvIII cells were dissociated into single cells and seeded into six‐well plates containing 3 mL serum‐free medium. Spheres were collected for protein extraction after 3 weeks. U87EGFRvIII and N33EGFRvIII cells were seeded in 96‐well plates containing 100 µL completed stem‐cell medium at different densities. 60 wells per plate were planted and 100, 50, 20, 10 cells per well. Each well was examined for the formation of tumor spheres after 3 weeks. The frequency was calculated using extreme limiting dilution analysis (http://bioinf.wehi.edu.au/software/elda/).

*Luciferase Reporter Assay*: GBM cells were planted in 12‐well plates, and were cotransfected with 100 ng RelA/p65 expression plasmid, 100 ng luciferase reporter vectors, and 20 ng pRL‐TK plasmids using Lipofectamine 3000 reagent (Invitrogen). After 24 h post‐transfection, cells were cultured in normal complete medium. Luciferase and renilla activities were detected by microplate reader.

*Immunofluorescence Assay*: Immunofluorescence assay was performed according to a previously described protocol.^29^ Briefly, cells were seeded on poly‐l‐lysine‐coated glass coverslips and treated with or without 200 × 10^−6^ m TMZ for 48 h. Following fixation in ice‐cold polyformaldehyde at 4 °C overnight, the cells were permeabilized with 0.3% Triton‐X100 for 5 min, blocked with 1% bovine serum albumin for 1 h at room temperature, and then stained with a γ‐H2AX‐specific primary antibody (Abcam, 1:200) and phalloidin (CST, 1:200) overnight at 4 °C. After washing three times, the primary antibody was detected by Alexa Fluor 488‐conjugated secondary antibodies (Molecular Probes/Life Technologies, Eugene, Oregon, USA, 1:100) for 2 h, and nuclei were stained by DAPI for 5 min at room temperature. Images were captured using an Olympus FluoView 1200 confocal microscope (Olympus, Tokyo, Japan). To objectively compare differences in immunofluorescence due to various treatments, all confocal scanning parameters were kept constant, and images were minimally processed to maintain data integrity.

*Intracranial Mouse Model*: All experimental protocols were conducted within Tianjin Medical University guidelines for animal research and were approved by Institutional Animal Care and Use Committee. 5 week old female nude mice, which were purchased from the Chinese Academy of Medical Science Cancer Institute, were used to establish intracranial GBM xenografts. Previously, U87 cells were infected with E2F6‐overexpressing lentivirus, and U87‐EGFRvIII cells were transduced with an E2F6 siRNA lentivirus. U87, U87+E2F6, U87‐EGFRvIII, and U87‐EGFRvIII+E2F6 KD cells labeled with luciferase were used for the mouse xenograft model. A total of 5 × 10^5^ cells were intracranially injected into each mouse. The mice were then treated with intraperitoneal injection of TMZ (5 mg kg^−1^ d^−1^) for 2 weeks at 5 days on/2 days off a week. Bioluminescence imaging was taken on days 7, 14, 21, and 28 to monitor intracranial tumor growth. The data were normalized to bioluminescence detected at the initiation of treatment for each animal. Patient‐derived GBM tumor samples were resected, minced, and suspended with ice‐cold PBS before centrifugation at 13 000 rpm for 5 min at room temperature. Then, the supernatants were discarded and the pellets were dispersed to separate cells with trypsin for 5 min. The cells were collected after being transduced by lentivirus expressing luciferase for 1h, and then a total of 5 × 10^4^ cells were intracranially injected into each mouse. A week later, mice were randomly divided into four groups (seven mice per group). The mice were then intraperitoneally injected with PBS, TMZ (5 mg kg^−1^ d^−1^), JSH‐23 (6 mg kg^−1^ d^−1^), or TMZ combined with JSH‐23 for 2 weeks at 5 days on/2 days off per week. Body weight was measured every 2 days until the mice succumbed to the disease. The error bars shown in the figures indicated standard deviation (SD). At the end of the experiment, Kaplan–Meier survival curves were plotted to show survival. After the whole treatment of TMZ and JSH‐23, the representative mice from each group were euthanized and the xenograft tumor was removed for immunohistochemistry analysis. Anti‐EGFRvIII (CST), anti‐p‐NF‐κB (p‐p65, CST), anti‐NF‐κB (p65, CST), and anti‐E2F6 (GeneTex) antibodies were used to detect the expression of each protein.

*Pathway Analysis*: Ingenuity Pathway Analysis (Qiagen, Redwood City, www.qiagen.com/ingenuity) was utilized to identify significant biological pathway(s) in both the CRISPR library and the RNA‐Seq datasets. A set of focus genes were used for the data input for both individual and canonical pathway analyses using *P* < 0.01 such that only significant genes were considered for significant pathways. Canonical pathway analysis was performed with the IPA library which contains most significant genes in canonical pathways. Networks were then algorithmically generated based on their connectivity. A Fisher's *t*‐test value of *P* < 0.05 was used to determine the statistical significance of a pathway.

*Statistical Analysis*: Statistical analysis was performed using SPSS software (version 22.0, SPSS Inc., Chicago, IL, USA). Data were presented as the mean ± SD of three independent experiments. *T*‐test was used to analyze the differences between two independent groups, and one‐way analysis of variance (ANOVA) with the post hoc least significant difference (LSD) test was used to analyze differences in more than two groups. Log‐rank tests were used to analyze significant differences between Kaplan–Meier survival curves using GraphPad Prism software. Cox regression was used to determine the prognostic value of each variable with PFS in CGGA GBM patients. Experiments were repeated at least three times. Values of *P* < 0.05 were considered statistically significant.

## Conflict of Interest

The authors declare no conflict of interest.

## Supporting information

Supplementary

Supplementary

Supplementary

Supplementary

Supplementary

Supplementary

Supplementary

Supplementary

Supplementary
