# Supplementary material for: Genome‐Wide CRISPR‐Cas9 Screening Identifies NF‐κB/E2F6 Responsible for EGFRvIII‐Associated Temozolomide Resistance in Glioblastoma
Source: Adv Sci (Weinh). 2019 Jul 24;6(17):1900782. doi: 10.1002/advs.201900782 (PMC6724471; doi:10.1002/advs.201900782)
Supplement: Supplementary file 1 — Supplementary [file ADVS-6-1900782-s001.pdf]

Copyright WILEY-VCH Verlag GmbH & Co. KGaA, 69469 Weinheim, Germany, 2019.

ADVANCED SCIENCE
Open Access

Supporting Information

for *Adv. Sci.,* DOI: 10.1002/advs.201900782

Genome-Wide CRISPR-Cas9 Screening Identifies NF-κB/E2F6 Responsible for EGFRvIII-Associated Temozolomide Resistance in Glioblastoma

Kai Huang, Xing Liu, Yansheng Li, Qixue Wang, Junhu Zhou, Yunfei Wang, Feng Dong, Chao Yang, Zhiyan Sun, Chuan Fang, Chaoyong Liu, Yanli Tan, Xudong Wu, Tao Jiang,* and Chunsheng Kang**

Copyright WILEY-VCH Verlag GmbH & Co. KGaA, 69469 Weinheim, Germany, 2019.

Supporting Information

Genome-wide CRISPR-Cas9 screening identifies NF-κB/E2F6 responsible for EGFRvIII-associated Temozolomide resistance in GBM

Kai Huang, Xing Liu, Yansheng Li, Qixue Wang, Junhu Zhou, Yunfei Wang, Feng Dong, Chao Yang, Zhiyan Sun, Chuan Fang, Chaoyong Liu, Yanli Tan, Xudong Wu, Tao Jiang,* Chunsheng Kang**

Supplemental Figures

Figure S1

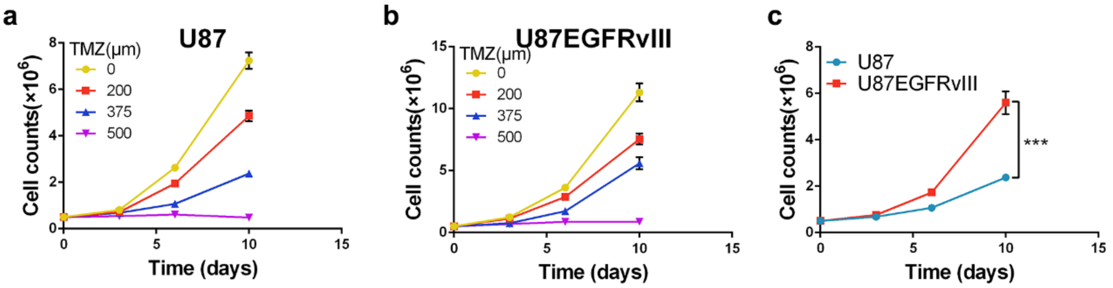

Supplemental Figure S1. U87EGFRvIII cells show more resistant to TMZ than U87 cells

(a-b) When treated with TMZ at 375 µM concentration, the growth rates of U87 and U87/EGFRvIII cells were prohibited by 60-70%, and when treated with 500 µM TMZ, the cells almost stopped growing.

(c) When treated with TMZ at 375 µM, U87/EGFRvIII cells grew much faster than U87 cells, indicating that U87/EGFRvIII cells are resistant to TMZ.

Figure S2

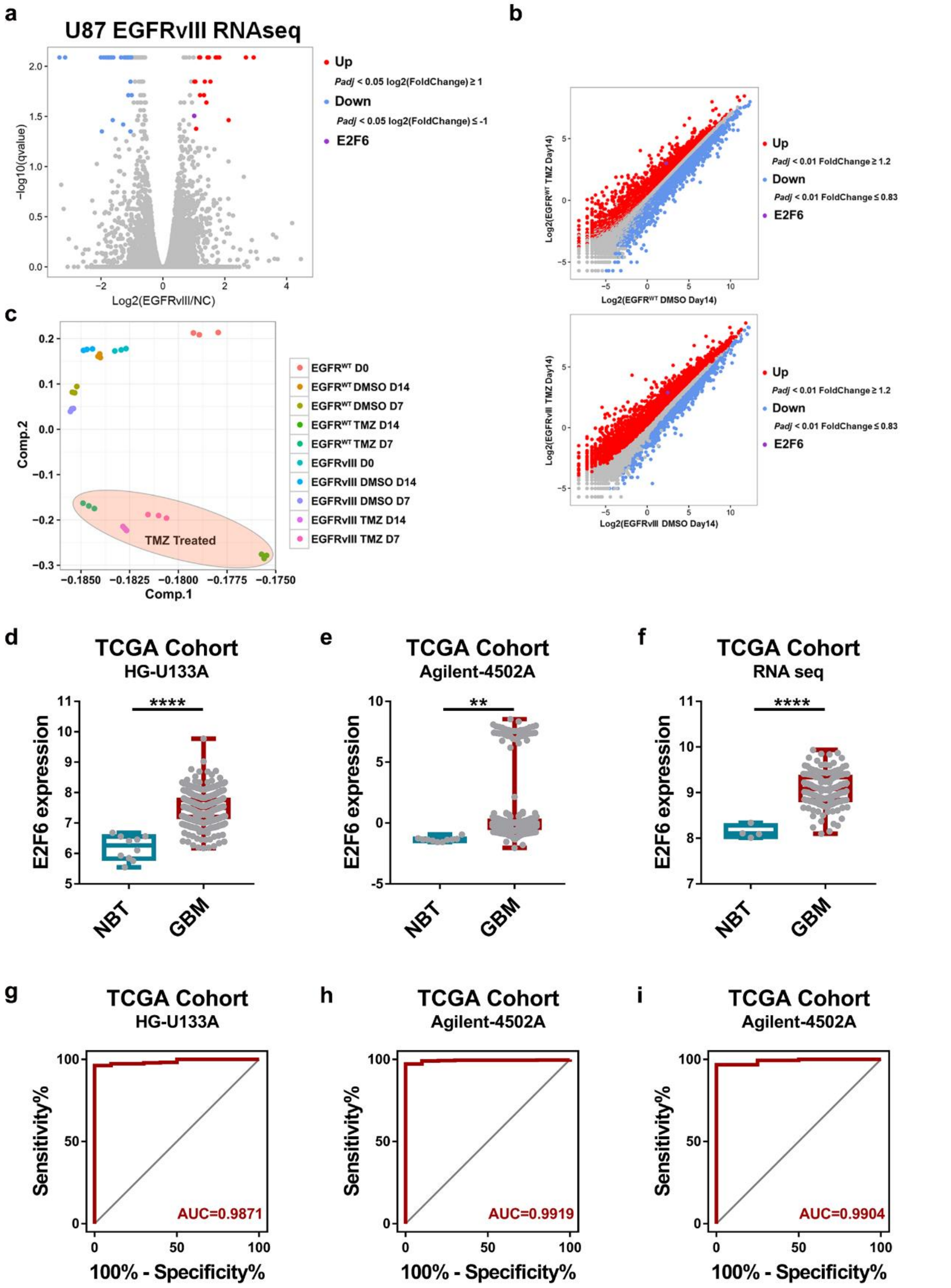

Supplemental Figure S2. E2F6 is a critical gene in TMZ-resistant GBM cells.

(a) RNA-seq analysis of U87 and U87EGFRvIII cells revealed up- and down-regulated genes in U87/EGFRvIII cells. E2F6 is an upregulated gene downstream EGFRvIII (Padj<0.05, log2 (fold change) ≥1 or ≤-1).

(b) CRISPR-Cas9 based whole-genome screening showed unregulation of E2F6 in U87 and U87EGFRvIII cells following TMZ treatment for 14 days.

(c) Principal component analysis (PCA) plot revealed the feasibility of discriminating cells by TMZ treatment.

(d-f) Significant elevated E2F6 in GBM as compared with normal brain tissue (NBT) was uncovered in the TCGA HG-U133A (d) and Agilent-4520A (e) microarrays and the RNA-seq (f) cohorts (**P < 0.01, ****P < 0.0001).

(g-i) The areas under the ROC curves indicated that E2F6 had an adequate diagnostic value for GBM patients.

Figure S3

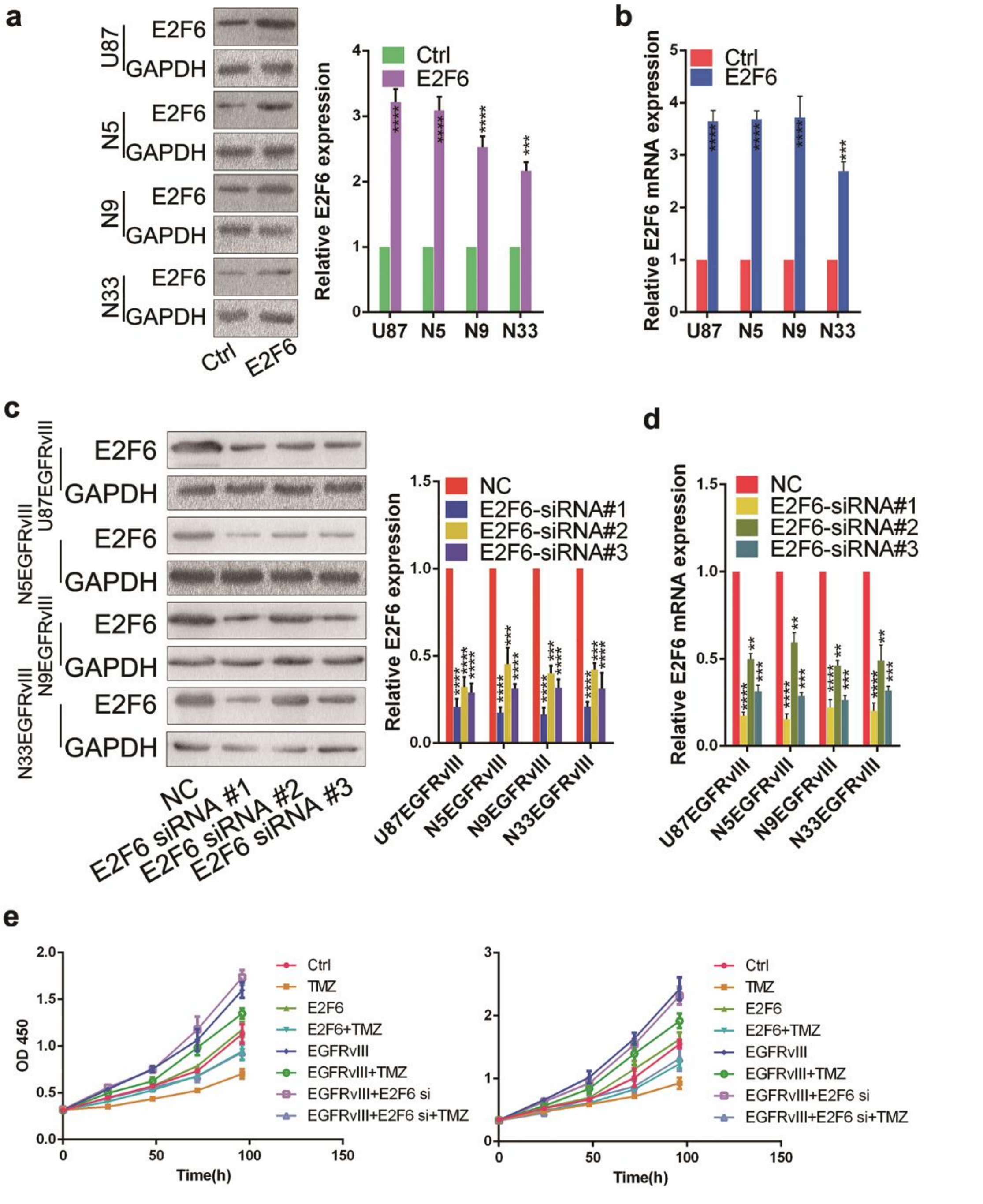

Supplemental Figure S3. E2F6 is a determinant in TMZ resistance in cell culture.

(a-b) Western blot (a) and qRT-PCR (b) analyses of E2F6 levels in GBM cells infected with lentiviruses expressing E2F6 or vector. GAPDH was served as a negative control. (***P < 0.001, ****P < 0.0001).

(c-d) Western blot (c) and qRT-PCR (d) analyses of E2F6 levels in indicated GBM

cells treated with E2F6 siRNA #1, E2F6 siRNA #2 and E2F6 siRNA #3 or control siRNA. E2F6 siRNA #1 showed the best E2F6 knockdown efficiency. (****P < 0.0001).

(e) Cell viability assay in 2 primary GBM cell lines (N9 and N33): Expression of E2F6 decreased whereas depletion of E2F6 increased TMZ sensitivity but had no effects on GBM cell proliferation.

Figure S4

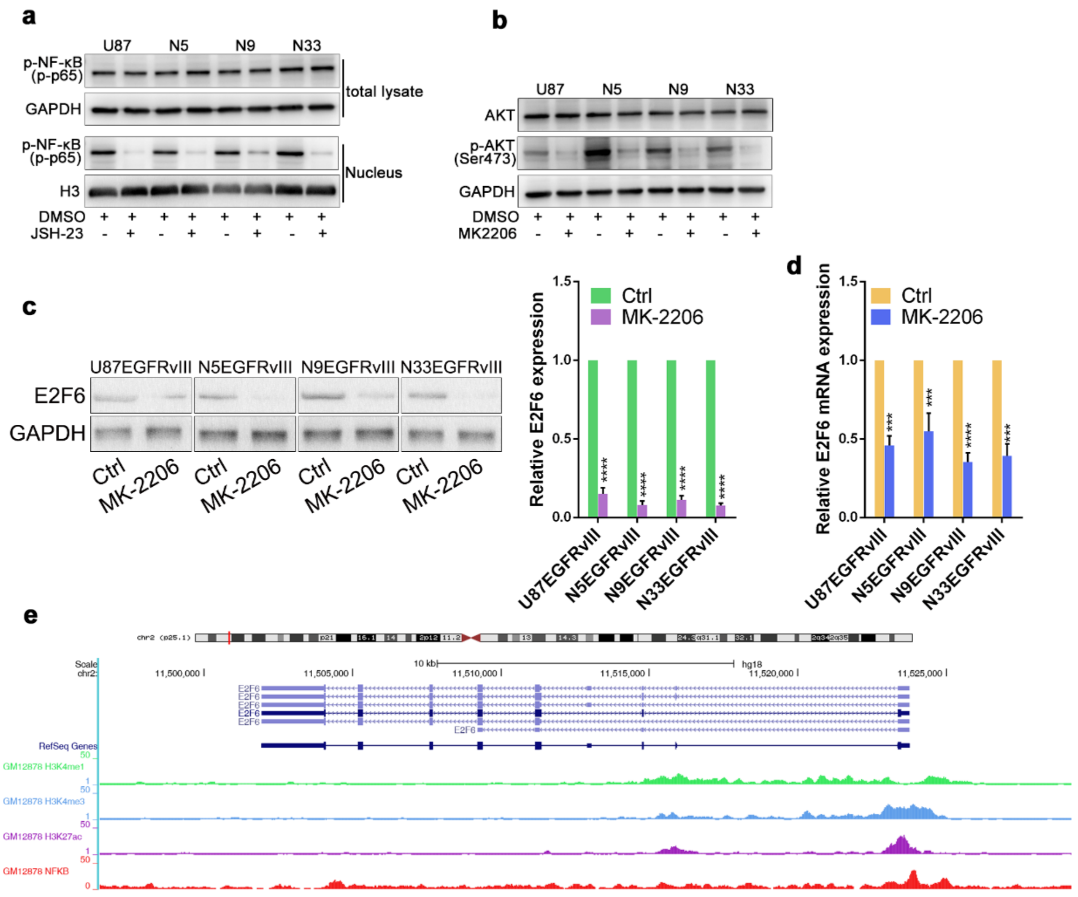

Supplemental Figure S4. E2F6 is upregulated by the EGFRvIII/PI3K/AKT/NF-κB cascade.

(a) The total lysate and the nuclear fraction were used to show p-NF-κB P65 levels in 4 GBM cell lines, after treatment with 10μM JSH-23 for 24 hours.

(b) Western blots were performed to analyze the levels of AKT and p-AKT (Ser 473) in total lysate of indicated cells after treatment with 10μM MK2206 for 48 hours.

(c-d) Immunoblotting (c) and qRT-PCR (d) analyses of E2F6 expression in 4 EGFRvIII-expressing GBM cell lines after treatment with and without MK-2206. GAPDH was served as the negative control. (***P < 0.001, ****P < 0.0001).

(e) ChIP-seq data from UCSC Genome Browser showed that H3K4me3, H3K27ac and NF-κB were enriched in the E2F6 promoter in GM12878 cells.

Figure S5

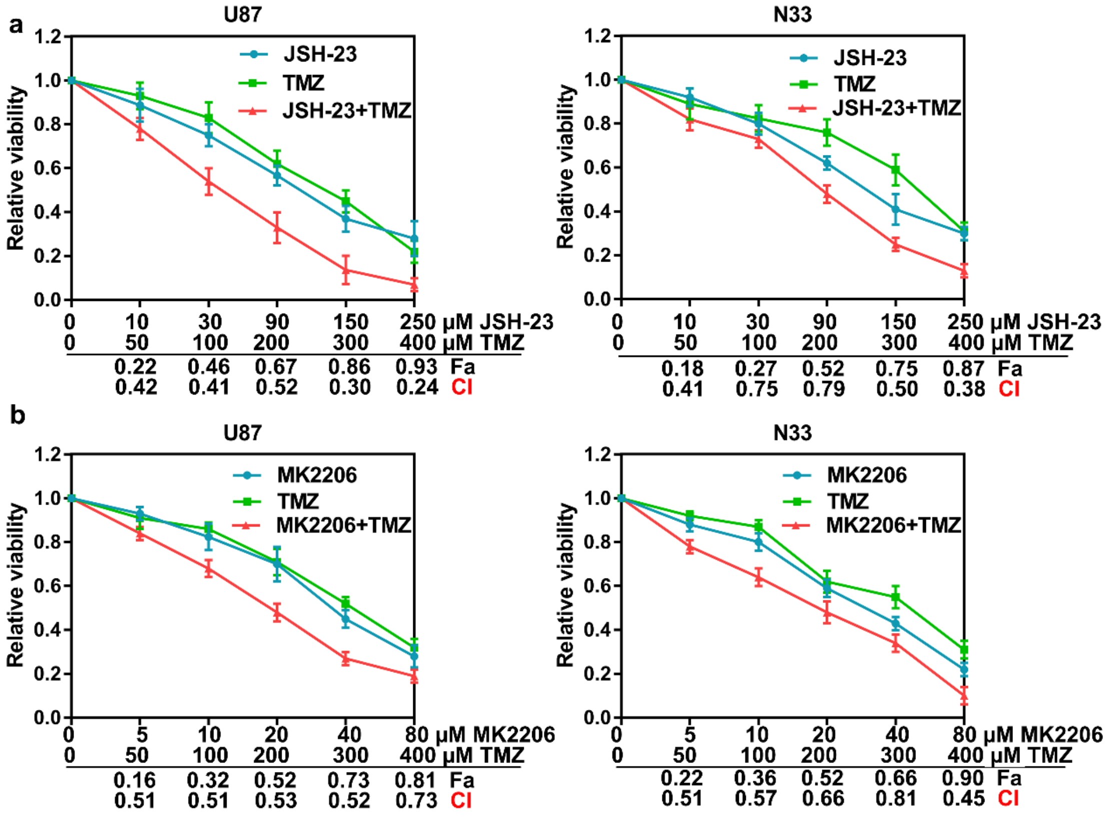

Supplemental Figure S5. NF-KB inhibitor (JSH-23) and p-AKT inhibitor (MK2206) both have synergistic effect with TMZ treatment.

(a) The cytotoxicity of JSH-23 and TMZ was detected by CCK-8. U87 and N33 cells were incubated with increasing concentrations of JSH-23 and TMZ alone or in combination for 48 h, and the cell viability was determined. The combination index (CI) and the fraction affected by the dose (Fa) values for the combination of JSH-23 and TMZ were calculated calculated using CalcuSyn software (Version 2; Biosoft). 0<CI<1 indicates a synergistic interaction. The averages and error bars represent the mean±s.d. from three independent experiments.

(b) The cytotoxicity of MK2206 and TMZ detected by CCK-8. As described in (a), MK2206 also had synergistic effect with TMZ treatment.

Table S1. Clinical Characteristics of 53 GBM patients in Tiantan Hospital

**Variables**	**E2F6 Low (n=34)**	**E2F6 High (n=19)**	***P* value**
**Age (median, years)**	43.5	46	*N.S.* [a]
**Sex**			
Female	13	10	*N.S.* [b]
Male	21	9	
**Positively Stained Cells**			
Median (Range)	2 (1-3)	3 (2-3)	<0.001 [a]
**The intensity of staining**			
Median (Range)	2 (1-2)	2 (2-3)	<0.0001 [a]
**E2F6 IHC Score**			
Median (Range)	2.5 (1-4)	6 (6-9)	<0.0001 [a]
**Progression-free survival**			
Median (days)	513.5	259	0.003 [a]

a, Student t test; b, Fisher's exact test.
